# TCR Affinity for Self-Ligands Influences the Development and Function of Encephalitogenic T Cells

**DOI:** 10.1371/journal.pone.0017702

**Published:** 2011-03-17

**Authors:** Jianwei Li, Omar Vandal, Derek B. Sant'Angelo

**Affiliations:** 1 Immunology Program, Sloan-Kettering Institute, Memorial Sloan-Kettering Cancer Center, New York, New York, United States of America; 2 Louis V. Gerstner Jr. Graduate School of Biomedical Sciences, Memorial Sloan-Kettering Cancer Center, New York, New York, United States of America; 3 Weill Graduate School of Medical Sciences, Cornell University, New York, New York, United States of America; Oklahoma Medical Research Foundation, United States of America

## Abstract

The specificity and affinity of self-reactive T cells is likely to impact the development of autoimmune-disease causing T cells in the thymus as well as their function in the periphery. We identified a naturally occurring, low affinity variant of an MBP Ac1-11/I-A^u^ specific TCR that is known to induce EAE. Thymocytes in mice carrying the transgenes for this low affinity TCR were poorly positively selected, as compared to their high affinity TCR expressing counterparts. Nonetheless, CD4 T cells bearing the low affinity TCR accumulated in the periphery of the mice. Unlike mice expressing the high affinity TCR, these mice very rarely developed disease. However, if endogenous TCR expression was eliminated by breeding to RAG1 deficient mice, 100% of the mice carrying either the high or the low affinity versions of the TCR developed EAE. Intriguingly, while the incidence of EAE increased, the age of onset of disease in both mice was identical. These data suggest disease onset occurs during a short window of mouse development.

## Introduction

Developing T cells require a functional interaction between the TCR and self-MHC:self-peptide complexes [1], [2]. In general, it is thought that low affinity interactions lead to maturation, whereas high affinity interactions result in clonal deletion. These complementary selection processes lead to the development of a self-referential T cell repertoire that is both tolerant to self, yet sufficiently diverse to respond to pathogenic challenges [3], [4], [5], [6], [7]. Data suggests that in addition to defining positive versus negative selection, TCR affinity for selecting ligands may also play an instructive role in defining lineage commitment. For example, high affinity interactions have been suggested to direct developing thymocytes towards functionally distinct lineages such Treg, iNKT, CD8αα and Ly6C^+^ T cells [1], [8], [9]. Additionally, commitment to the conventional CD4 and CD8 lineages may also be influenced by TCR affinity [10].

EAE is an inflammatory, demyelinating disease manifested by acute, chronic or relapsing paralysis [11], similar to human multiple sclerosis [12]. The disease is mediated primarily by MHC class II-restricted CD4^+^ T cells that secrete inflammatory cytokines [11]. Stimulation of anti-inflammatory Th2 T cells that secrete IL-4 and IL-10, however, has been shown to inhibit EAE [13]. To determine if TCR affinity influences the onset of EAE, we identified and cloned a TCR that has considerably lower affinity for the Ac1-11 MBP peptide presented by I-A^u^ as compared to a previously published TCR [14]. Transgenic mice expressing this new low affinity TCR were generated and compared to an existing high affinity TCR transgenic mouse.

On a wild type B10.PL background, the incidence of spontaneous disease initiated by T cells expressing the low affinity TCR was very low, as compared to the high affinity TCR transgenic mouse. When bred to a RAG1 deficient background, which is known to increase the frequency of EAE, at least in part due to the loss of residual CD4^+^CD25^+^ Tregs [15], [16], [17], both the high and low affinity TCRs induced the onset of EAE in 100% of mice. Intriguingly, the age of onset of disease in mice with and without Tregs was nearly identical.

## Materials and Methods

### Animals

All animal work was done in compliance with Memorial Sloan-Kettering Cancer Center's Internal Animal Care and Use Committee (IACUC) and the guidelines of the Federal Office of Laboratory Animal Welfare. The approved protocol number was 05-03-005. Mice were housed and bred in specific pathogen-free environments in microisolators in the Research Animal Resources Center (RARC) of Memorial Sloan-Kettering Cancer Center (MSKCC). Additional mice were purchased from The Jackson Laboratory. Animal housing rooms were under temperature and humidity control, the mice were not subjected to water or food restrictions and bedding material was placed in each cage. Four full time veterinarians and six veterinarian technicians staff the facility. The veterinary staff is located on site and a clinical veterinarian is available at all times. Animal care staff carried out routine husbandry procedures including changing cages, feeding and watering. All mice were sacrificed prior to use. Euthanasia was conducted in accordance with the American Veterinary Medical Association (AVMA) Guidelines on Euthanasia. Briefly, mice were sacrificed by asphyxiation with CO2. CO2 is delivered into the cages at less than 5psi per second. Death of the animal is confirmed by lack of respiration and toe pinch. CO2 euthanasia stations are inspected regularly by IACUC personnel. After confirmation of death, tissues were removed for experiments. In studies involving the incidence of spontaneous EAE, mice were examined at least three times per week for evidence of disease. Histological analysis confirmed pathological lesions associated with EAE in hindlimb-paralyzed mice (data not shown). Of note were mononuclear cell accumulations in the perivascular, meningeal and parenchymal regions of the brain.

### Hybridomas

TCR Vα2 depleted or total T cells (see results section) were activated and fused to BW5147 cells as previously described [18]. Fused cells were plated at a density likely to generate clonal populations. In addition, hybridomas used for assays were cloned by limiting dilution and analyzed by FACS for TCR and CD4 expression. Stimulation assays were carried out as previously described [18].

### TCRα transgene

cDNA made from the hybridomas was screened with a panel of 24 Vα specific primers that we generated based on published sequences [19]. The primers were tested by amplifying cDNA generated from mRNA from wild type thymocytes RNA. PCR products were cloned (TOPO TA, Invitrogen) and sequenced. Based on this information, PCR primers were designed to amplify genomic DNA from one of the Vα6 hybridomas from 5′ of the ATG to 150 bp 3′ of the end of the Ja segment (within the Jα-Cα intron). This DNA fragment was cloned, sequenced and then transferred into the pαTris vector [20]. The purified transgene was injected into fertilized F1 eggs obtained by mating B10.PL and C57Bl/6 mice. Transgene positive founders were backcrossed to B10.PL and typed to confirm homozygous expression of I-A^u^. Two different founder lines were established. Data from the two founder mouse lines (FACS, onset of EAE, etc.) was nearly identical and, therefore, only one founder was fully analyzed.

### T Cell Proliferation Assays

Lymph nodes and spleens from healthy 6-8 week-old MBPαβ TCR and OVαβ TCR transgenic mice were harvested. Magnetic depletion (BioMag beads; PerSeptive Biosystems, Framingham, MA) using antibodies against MHC class II, CD8, Mac1 and NK1.1 was used to enrich for CD4^+^ T cells. Cell purity (typically >90%) was checked by FACS with antibodies against CD4 and TCR Vβ8. APCs were prepared by incubation of single-cell suspensions of B10.PL splenocytes with antibodies against CD4, CD8, and Thy 1 followed by incubation with rabbit complement and mitomycin C for 30 min at 37°C. Experiments were set up in triplicate in 96-well, round-bottom plates with 2.5×10^4^ T cells (total cell number adjusted based upon purity) and 1×10^5^ APCs. Assays were pulsed with 0.5 µCi per well of [3H]thymidine after 48 hours and were harvested after an additional 24 hours. Blocking assays were carried out as previously published [21]. No T cell proliferation is induced by the F23.1 antibody at the doses that were used.

### Tissue preparation and FACS Analysis

Single-cell suspensions were prepared by dissociation of tissues between glass slides. To block nonspecific antibody binding, cells were first incubated for 15 minutes with anti-mouse FC-RIII antibody and normal mouse serum (Jackson Labs). Cells were washed and then stained for 30 minutes with the appropriate cocktail of the following antibodies from BD Biosciences: CD4-PerCP-Cy5.5, CD8-APC, Vβ8 FITC, Vα2-PE, CD69-PerCP-Cy5.5, CD44-FITC, CD25-PE, CD62L-APC and TCRCα-APC. Cells were washed and analyzed on a Becton Dickinson LSR or DakoCytomation Cyan.

### Cell sorting

Cell sorts were done in MSKCC's Flow Cytometry Core Facility on a DakoCytomation MoFlo. Sorted populations were reanalyzed and proved to be over 98% pure.

## Results

### Identification of a low affinity TCR

Mice carrying the transgenes for a MBP AC1-11 peptide specific TCR (Vα2.3-Jα11; Vβ8.2-Jβ2.6) cloned from the 172.10 hybridoma have been described [14]. T cells from these TCR transgenic mice respond strongly to the Ac1-11 myelin basic protein (Ac1-11) peptide bound to I-A^u^ and these mice spontaneously develop EAE (1, 9). Previously, we have shown that mice carrying only the TCRβ transgene (MBPβ TCR) are highly enriched (approximately 0.01% of total CD4 SP thymocytes) for T cells specific for the Ac1-11:I-A^u^ complex [22]. As a result, in vitro culture of total lymph node cells taken from MBPβ TCR transgenic mice with the Ac1-11 peptide results in a strong, dose-dependent T cell response. Other than the somewhat restricted TCR repertoire, MBPβ TCR transgenic mice are indistinguishable from wild type mice since they have normal numbers of both CD4 and CD8 T cells that express a diverse TCR repertoire [22].

**Figure 1:**
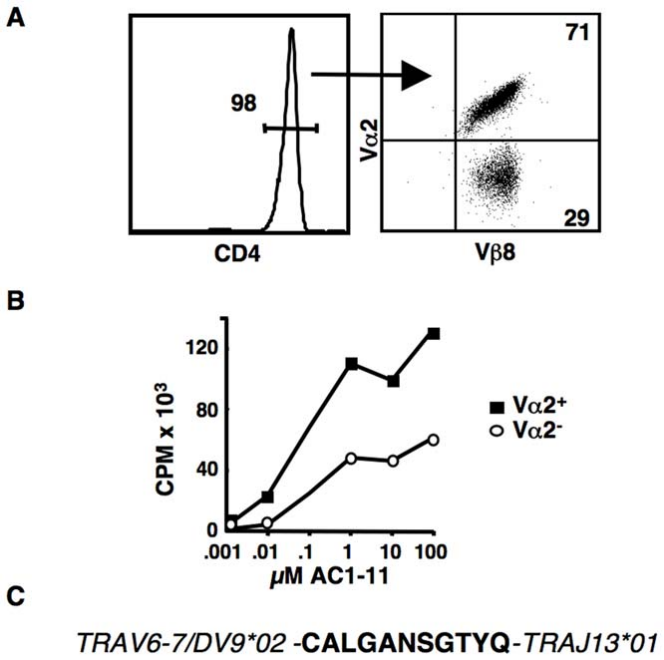
Analysis of lymphocytes from MBPβ transgenic mice cultured with the AC1-11 peptide. Total lymphocytes from MBPβ-only transgenic mice were bulk cultured with the MBP-derived AC1-11 peptide. Several additional rounds of activation were done by culturing with T cell depleted B10.PL splenocytes loaded with the AC1-11 peptide. FACS was carried out seven days after the final activation. (**A**) Nearly all remaining cells were CD4^+^ and expressed the transgene-encoded Vβ8 TCRβ chain. Across several experiments, approximately 30% of the T cells were not Vα2 positive. (**B**) The Vα2^+^ and Vα2^−^ T cells were sorted and then stimulated by the addition of irradiated, T cell depleted B10.PL splenocytes and titrated amounts of the AC1-11 peptide. Cells were pulsed with 3[H]thymidine after 48 hours and harvested after a total of 72 hours. Approximately 5×10^4^ T cells and 1×10^5^ APCs were used per well. Assays were carried out in triplicate and averaged. Results shown are representative of three independent experiments. (**C**) Predicted amino acid sequence of the CDR3α region of the Vα6 (TRAV6-7/DV9*02) – Jα13 (TRAJ13*01) TCR cloned from the Vα2^−^ population. Gene nomeclature based on the IMTG Marie-Paule convention [46].

In an effort to identify T cells with a low affinity for the MBP Ac1-11:I-A^u^ ligand, we harvested lymph node cells from MBPβ TCR transgenic mice and bulk cultured the cells with 10 µM of the peptide. After seven days, cells were analyzed by FACS using mAbs against CD4, Vβ8 and Vα2. As expected, nearly all of the surviving cells were CD4^+^ (Fig. 1A) and expressed that the transgene encoded Vβ8^+^ TCRβ chain (Fig. 1A). Interestingly, in six independent experiments, we consistently observed two different T cell populations when we stained with an anti-Vα2 mAb. Approximately 70% of the T cells were always found to be Vα2^+^, while approximately 30% of the cells were Vα2 negative (Fig. 1A). This ∼2∶1 ratio was found with several different doses of peptide and was stable if the T cells were activated several more times (data not shown).

Our finding that the non-Vα2 T cells were consistently a smaller percentage of the total cultured T cell population suggested that these cells might be expressing TCRs that were lower affinity as compared to the Vα2^+^ cells. To test this, we sorted the two populations from bulk cultured T cells that had gone through several rounds of activation in vitro. The sorted T cells were stimulated with titrated amounts of the Ac1-11 peptide presented by T cell depleted, irradiated B10.PL spleen cells. As shown, in Fig. 1B, the Vα2^+^ cells responded substantially better than their Vα2^−^ counterparts. While the magnitude of the T cell response varied in repeats of this experiment due to variability in the total cell numbers used, the difference in the response between the two T cell populations was consistent. Importantly, the anti-Vα2 mAb itself does not induce or block activation of T cells and, therefore, is not a factor in these T cell assays [23].

In an effort to clone the TCRα chain or chains used by the Vα2^+^ and Vα2^−^ populations, we generated T cell hybridomas [18]. For our first fusion, we used T cells that had been bulk cultured by feeding several times with irradiated B10.PL splenocytes loaded with 10 µM Ac1-10 peptide. Prior to the final activation, the bulk culture was split and magnetic beads were used to deplete out the Vα2^+^ cells from one of the two cultures. Two days after activation, the T cells were fused to BW5147 and plated at a density likely to produce single cell clones [18]. The resulting hybridomas were screened by FACS with mAbs against the TCRβ chain, Vα2 and CD4. Specificity of the clones was confirmed by carrying out T cells assays with Ac1-11 peptide loaded APCs. Activated T hybridomas produced IL-2, which was detected by the IL-2 dependent cell line, CTLL-2 (data not shown). Dose-response assays confirmed that, similar to the sorted T cells, the Vα2^+^ hybridomas responded much better to the Ac1-11 peptide (data not shown).

Twenty Vα2^+^, CD4^+^ clones and twenty Vα2^−^, CD4^−^ clones were selected and used for further studies. cDNA from the Vα2^+^ clones was PCR amplified with primers specific for Vα2 and the Cα gene segments. The amino acid sequences deduced from the sequences of the PCR products were identical both to each other and to the original Ac1-11/I-A^u^ specific 172.10 hybridoma. This finding is consistent with our previous work in which we found that positive selection in the thymus resulted in a repertoire that was enriched for TCRs using the TCRα chain found in the original 172.10 T cell hybridoma [22].

The Vα2^−^ hybridomas were also negative for the three other available anti-Vα gene segment mAbs. Therefore, we screened the clones by RT-PCR utilizing a panel of 24 different Vα specific primers that we designed to amplify all known mouse Vα gene segments. Other than the endogenous TCRα chain known to be expressed by the BW5147 cell line, only one primer set consistently resulted in a PCR product. We cloned and sequenced the PCR fragment and found that all twenty hybridomas expressed the identical TRAV6-7/DV9*02 - TRAJ13*01 (Vα6-Jα13) TCRα chain (Fig. 1C). Interestingly, other than a single amino acid change in the CDR3α segment, the TCRα chain we cloned is identical to a TCRα chain cloned from an AC1-9/I-Au specific T cell derived from a wild type B10.PL mouse [24]. This closely related TCR, however responds strongly to the AC1-9/peptide:MHC complex [24].

These data suggested that T cells from MBPβ TCR transgenic mice bearing one of only two different TCRs could respond to the Ac1-11/I-Au complex. We were concerned, however, that other T cells expressing different TCRs might have been lost during the extended culturing of these cells. Therefore, we generated a new set of hybridomas following a single stimulation of total lymph nodes cells harvested from the MBPβ TCR mice. The hybridomas were screened with our panel of 24 Vα-specific primers, as described above. cDNA was generated from approximately 20 Vα2^+^ and Vα2^−^ clones, PCR amplified and sequenced. Once again, we were only able to identify the same two TCRs. Therefore, under these conditions, only T cells expressing one of the two different TCRs responded to the Ac1-11 peptide presented by I-A^u^ APCs. Importantly, the Vα6-Jα13 TCR, which we named the OVαβ TCR, responded less well to this antigen (Fig. 1B and data not shown) and, therefore, can be considered a functionally lower affinity TCR.

### Inefficient positive selection of thymocytes expressing the low affinity OVαβ TCR

To analyze the in vivo function of T cells expressing the low affinity OVαβ TCR, we generated TCR transgenic mice. The original MBP Ac1-11 specific transgenic mice (MBPαβ) were generated as single-chain transgenics (TCRα and TCRβ were not coinjected). Therefore, it was only necessary for us to construct the OVα TCR transgene. A 650 bp fragment containing the Vα6-Jα13 gene segment was generated by PCR of DNA from one of the hybridomas described above. The PCR product was cloned, sequenced and then transferred into the ∼20 kb pTα cassette vector [20]. This vector contains a TCR Vα promoter and the complete TCR Cα gene, including the Cα enhancer and LCR. Overall, this transgene vector is very similar to the cosmid vector used for generating the MBPα transgene [14] and, therefore, it is expected that the two transgenic constructs should behave similarly in vivo. The purified transgene was microinjected into F1 blastocysts obtained from B10.PL x C57Bl/6 female mice.

Two founder transgenic mice were identified and backcrossed to B10.PL mice that carried the transgene for the MBPβ TCR chain. The phenotype of the two founder lines was nearly identical and, therefore, data from only one line is presented. Mice carrying the transgenes for both the OVα TCR chain and the MBPβ TCR chain as well as homozygous for the H-2u MHC haplotype were selected for analysis. Negative littermates, MBPβ TCR only littermates and age matched MBPαβ TCR transgenic mice were used for controls. In addition to the direct injection into B10.PL x C57Bl/6 F1 blastocysts, mice were backcrossed to B10.PL for at least five generations.

**Figure 2:**
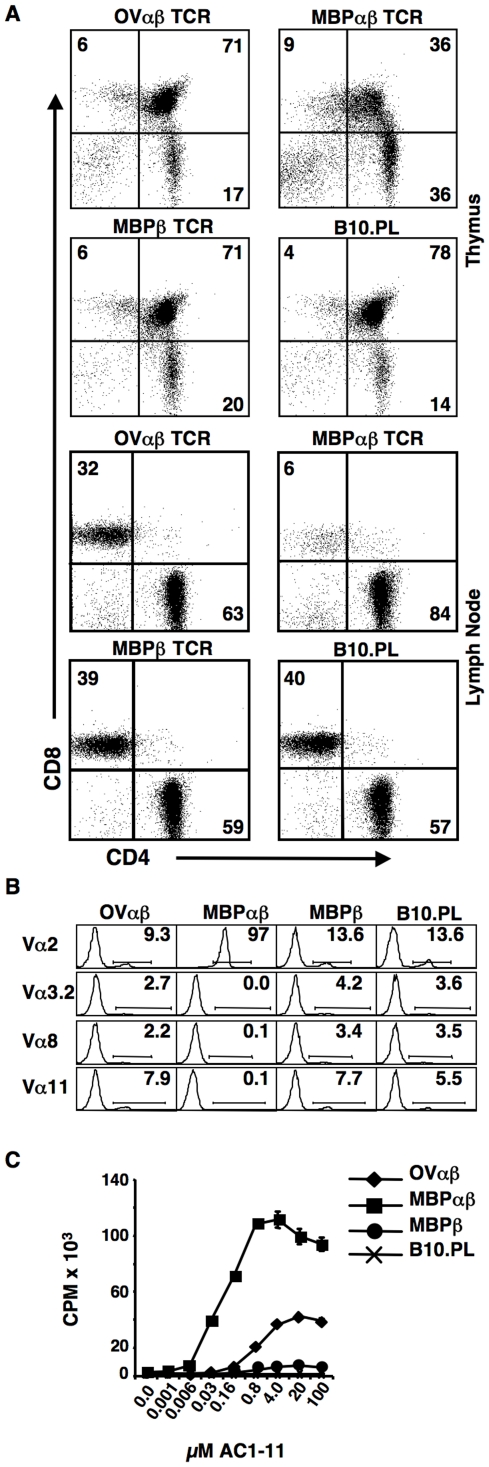
Development of OVαβ TCR expressing thymocytes. The strong skewing of thymocytes expressing the high affinity MBPαβ TCR towards the CD4 lineage is not seen in OVαβTCR expressing mice. Expression of only the MBPβ chain does not markedly alter T cell development. (**A**) Single cell suspensions of thymocytes (top) and lymph node cells (bottom) from OVαβ, MBPαβ, MBPβ transgenic and transgene negative mice were stained with antibodies against CD4, CD8 and TCR Cβ. Only TCR Cβ^+^ cells are shown in the lymph node cell plots. The number indicates the percent of cells in the quadrant. Only subtle variations in the percent of cells within each quadrant were noted during repeat experiments. (**B**) CD4 SP thymocytes from OVαβ TCR transgenic mice express a high percentage of endogenous TCRα chains, whereas MBPαβ TCR transgenic mice predominantly express the transgene encoded Vα2 chain. Single cell suspensions of thymocytes from the indicated mouse strain were stained with antibodies against CD4, CD8 and individually with each of the four available TCR Vα specific antibodies. The percent of cells within the CD4 single positive population expressing each of the Vαs is shown. More than 1×10^6^ events were collected per sample. One of two experiments is shown. Greater than 98% of T cells in the OVαβ, MBPαβ and MBPβ mice expressed the transgene encoded Vβ8.2 TCR. (**C**) Increased levels of proliferation in response to the Ac1-11 peptide indicate a substantial number of antigen specific T cells develop in the OVαβ mice. CD4 T cells were enriched by negative selection. Double cultures were set up with 2.5×10^4^ CD4^+^ T cells and 1×10^5^ T cell depleted, irradiated B10.PL splenocytes plus the indicated amount of the MBP Ac1-11 peptide. Assays were pulsed with 3[H]thymidine after 48 hours and harvested after an additional 24 hours. Each condition was set up in triplicate and data was averaged. One example of three experiments is shown.

The increased percentage (consistently over 30%) of CD4 single positive (CD4SP) thymocytes expressing the MBPαβ TCR as compared to nontransgenic littermates or littermates carrying only the MBPβ TCR chain indicated strong positive selection of this TCR (Fig. 2A). In sharp contrast, thymocytes from mice expressing the OVαβ TCR had little obvious skewing towards the CD4 lineage (Fig. 2A). Indeed, the percentage of cells in the various CD4 versus CD8 quadrants identified by FACS was very similar to what was seen for MBPβ TCR only and wildtype mice (Fig. 2A). Overall cellularity of the thymuses from the OVαβ TCR and MBPαβ TCR mice was similar, although, as with all TCRαβ transgenic mice, the cellularity was reduced as compared to TCRβ only and wild type mice. Finally, in the periphery, in sharp contrast with the ∼15∶1 CD4:CD8 ratio of MBP T cells (Fig. 2), the CD4:CD8 ratio of T cells from the OVαβ TCR mice was only ∼2∶1, similar to the MBPβ only TCR transgenic mice.

Most likely the failure of the MHC class II restricted OVαβ TCR to increase overall percentages of CD4SP thymocytes and CD4^+^ T cells was due to poor positive selection. Inefficient positive selection has been shown to result in the replacement of transgenic TCRα chains with α chains from the endogenous locus [25], [26]. It has also been shown that a rearranged VαJα gene segment that was introduced into the genomic Jα region was removed by secondary TCRα rearrangements when the selecting MHC allele was not present [27], [28]. We were not able to directly measure the loss of cell surface expression of the transgene encoded TCRα chain since a mAb specific for this Vα gene segment does not exist and a published I-A^u^:Ac1-11 tetramer was no longer available. Therefore, to estimate the loss of the transgene-encoded TCRα, we stained thymocytes from OVαβ TCR, MBPαβ TCR, MBPβ TCR and wild type B10.PL mice with anti-TCR Vα2, Vα3.2, Vα8 and Vα11 mAbs (Fig. 2B). In the MBPαβ TCR mice, over 97% of the CD4SP thymocytes expressed the transgene encoded Vα2 TCR (Fig. 2B), while the other Vαs were hardly detectable. In contrast, CD4SP thymocytes from the OVαβ TCR mice (Fig. 2B) expressed high levels of all four TCR α chains in CD4 single positive thymocytes (9.3% Vα2, 2.7% Vα3.2, 2.2% Vα8 and 7.9% Vα11) (Fig. 2B). Indeed, the percentage of CD4SP thymocytes expressing each endogenous Vα was similar to what was seen in MBPb TCR only and wild type B10.PL mice (Fig. 2B). These data strongly suggest that that thymocytes carrying the transgene expressing the low-affinity OVαβ TCR commonly revised their TCR and, as a consequence, expressed endogenously rearranged TCRα chains.

To directly test for the presence of AC1-11 specific T cells in the OVαβ TCR mice, we purified CD4 T cells from the spleen and lymph nodes of OVαβ TCR, MBPαβ TCR, MBPβ TCR only transgenic mice as well as from control transgene negative mice. T cell proliferation assays were carried out by incubating CD4^+^ T cells with irradiated B10.PL splenocytes plus titrated amounts of the AC1-11 peptide (Fig. 2C). As anticipated, purified MBPαβ TCR T cells proliferated strongly, while wild type CD4 T cells did not respond at all. MBPβ TCR CD4 T cells also responded due to a relatively high frequency of antigen specific T cells, as previously published [22]. The CD4^+^ OVαβ T cells clearly responded much more vigorously than the MBPβ TCR only T cells. Therefore, although we did not observe strong skewing towards the CD4 lineage in mice carrying the OVαβ TCR transgenes, we were able to detect a relatively strong antigen-specific T cell response.

### Selection of MBPαβ TCR and OVαβ TCR expressing thymocytes in the absence of endogenous TCRs

**Figure 3:**
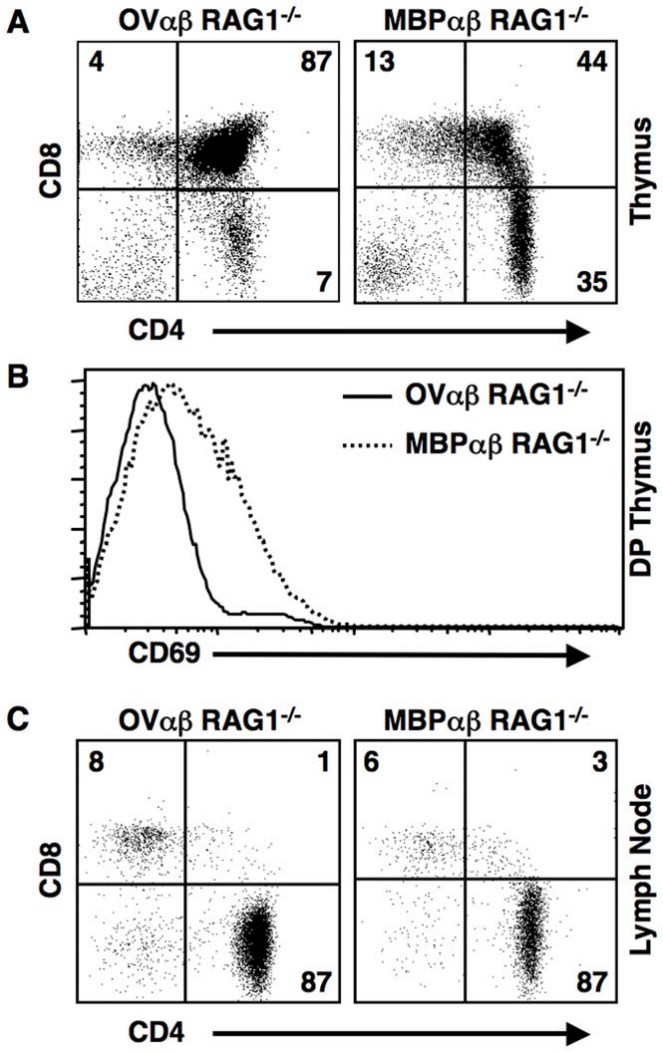
Thymocytes expressing the OVαβ TCR are poorly positively selected even in the absence of endogenous TCRs. (**A**) As compared to thymocytes expressing the high affinity MPBαβ TCR, the OVαβ TCR is very inefficiently positively selected, which is reflected in a much lower percentage of CD4SP thymocytes. (**B**) OVαβ TCR transgenic double positive (DP) thymocytes have a minimal upregulaton of CD69, which is an indicator of productive TCR-MHC interactions. (**C**) FACS analysis of lymph node cells shows that, despite poor positive selection, CD4 T cells accumulate in the OVαβ TCR transgenic mouse.

To exclude the T cells bearing endogenous TCRs, we generated B10.PL, RAG1 deficient mice carrying the transgenes for either the OVαβ TCR or the MBPαβ TCR. Similar to the data from the RAG sufficient mice, most MBPαβ TCR expressing thymocytes (Fig. 3A) were found to be at the CD4SP positive stage or had downregulated CD4 and CD8 and were likely on their way towards becoming CD4SP [22], [29]. In contrast, over 85% of the OVαβ TCR expressing thymocytes were found to be at the DP stage, with only 7% progressing to the mature CD4SP stage. Although the TCR is MHCII restricted, a small number of CD8^+^ cells are seen in both mice. Such “mismatched” cells are commonly found in the TCR transgenic mice.

Productive interactions of TCRs expressed by developing thymocytes with self-MHC:self-peptide complexes can be measured by staining for the upregulation of the early activation marker CD69 [29]. The minimal upregulation of CD69 on double positive (DP) thymocytes expressing the OVαβ TCR, as compared to the MBPαβ TCR, confirms that OVαβ TCR expressing thymocytes are very poorly positively selected (Fig. 3B). These data also ruled out increased negative selection of the OVαβ TCR expressing thymocytes since negative selection would also result in increased CD69 levels.

Despite poor positive selection, OVαβ TCR transgenic T cells accumulate in the periphery (Fig. 3C) in RAG1 deficient mice. Similar to the MBPαβ TCR T cells, nearly all of the OVαβ TCR expressing T cells are found within the CD4^+^ compartment. Unlike the CD4:CD8 ratio of the peripheral T cells from OVαβ TCR B10.PL mice, which was about 2∶1, the OVαβ TCR Rag1^−/−^ had a CD4:CD8 ratio greater than 10∶1, similar to what was seen in MBPαβ TCR Rag1^−/−^ mice (Fig. 3C). Spleen and lymph node cellularity was, on average, higher in the MBPαβ TCR RAG1 deficient mice.

### OVαβ TCR T cells have a lower affinity than MBPαβ TCR T cells for the I-A^u^:Ac1-11 complex

The original T cell cultures and the hybridomas generated from these T cells both showed that the OVαβ TCR had a functionally lower affinity for the I-A^u^:Ac1-11 complex than the MBPαβ TCR. Before evaluating in vivo function, it was necessary to determine if the naïve T cells collected from the two TCR transgenic mice retained this functional difference. Furthermore, by using a blocking mAb it was possible to directly compare the relative avidities of the two TCRs [21].

**Figure 4:**
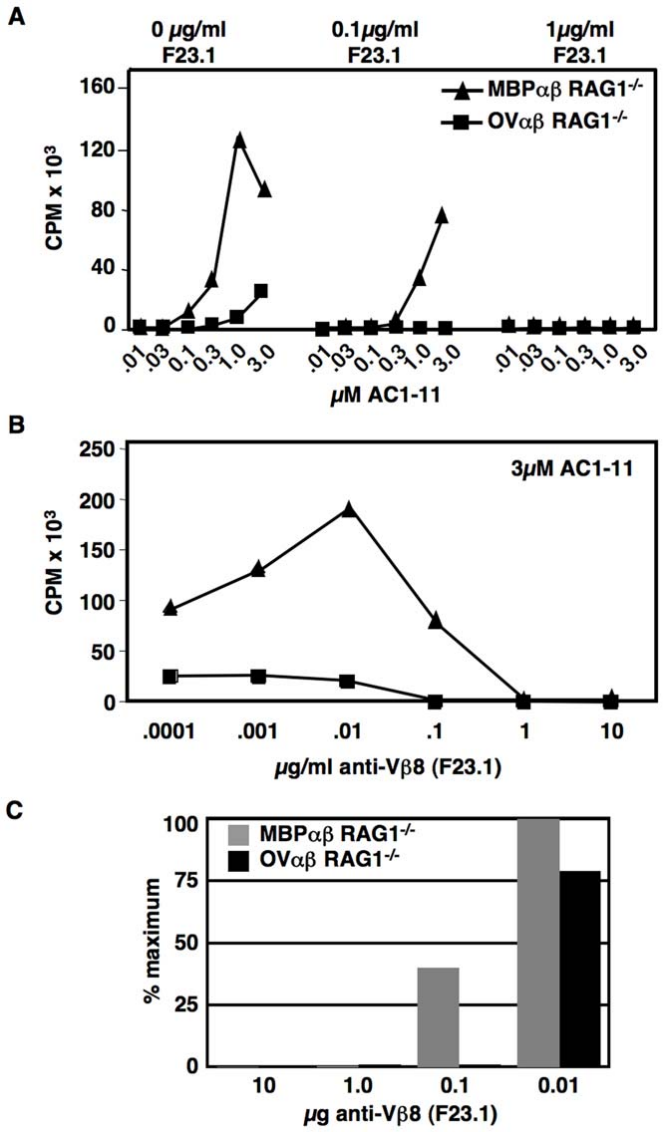
The relative affinity of the OVαβ and MBPαβ TCRs. Antibody blocking studies show that the OVαβ TCR is of much lower affinity for the I-A^u^:Ac1-11 peptide ligand as compared to the MBPαβ TCR. (**A**) Purified CD4^+^ T cells from MBPαβ TCR RAG1^−/−^ and OVαβ TCR RAG1^−/−^ mice were incubated with the indicated amount of antibody against Vβ8 (F23.1) and then challenged with titrated amounts of the Ac1-11 peptide presenting by B10.PL APCs. (**B**) Purified T cells were activated with 3uM Ac1-11, presented by B10.PL APCS, in the presence of titrated amounts of blocking antibody. Assays were pulsed after 48 hours and harvested at 72 hours. Each condition was done in triplicate and the data was averaged. One representative experiment of three is shown. (**C**) For clarity, the data from (B) are presented as the percent of the maximal response of the T cells without the blocking antibody.

CD4 T cells from OVαβ and MBPαβ TCR transgenic RAG1^−/−^ mice were purified by negative selection and then challenged with irradiated I-A^u^ splenocytes loaded with titrated amounts of the Ac1-11 MBP peptide. As we found with the original T cell lines as well as with the hybridomas, naïve OVαβ T cells respond much less well to the I-A^u^:Ac1-11 complex than the MBP TCR expressing T cells (Fig. 4A). To test the relative affinity of the two TCRs for I-A^u^:Ac1-11 complex, we first incubated purified T cells with different doses of F23.1 [30], an anti-Vβ8.2 mAb that we have previously shown does not cause activation of T cells at the concentrations used for these experiments [21]. We then examined the response to titrated doses of peptide. As can be seen in Fig. 4A, 1 µg/ml of F23.1 completely blocked the response of T cells from both mice. At a 10-fold lower dose of blocking mAb, however, MBPαβ T cells respond strongly, while OVαβ T cells are still completely blocked. We next used titrated amounts of the blocking mAb, while keeping the peptide concentration constant (Fig. 4B). Again, the response of T cells from the OVαβ mouse line was blocked by much less mAb. Since the absolute level of proliferation greatly differed between the two T cell types, we also expressed the data as the percent of the maximum response (Fig. 4C). Here it is clear that even just 10ngs of the anti-Vβ8.2 mAb is sufficient to partially block the response of the OVαβ T cells, whereas the MBPαβ T cells are not affected. Together these data show that the affinity of the OVαβ TCR for the I-A^u^:Ac1-11 complex is substantially less than the affinity of the MBP TCR for the same ligand.

### Development of spontaneous EAE in mice transgenic for low-affinity MBP-specific TCR

**Figure 5:**
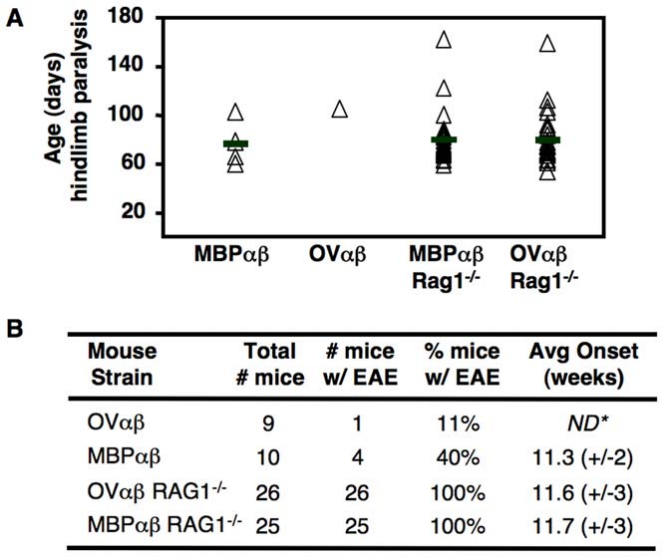
Development of spontaneous EAE in TCR transgenic mice. (**A**) RAG1 deficient mice were monitored for twelve months for overt signs of EAE (hind leg paraparesis). The day of the onset of EAE in individual mice is indicated on the chart. (**B**) More than 60% of MBPαβ mice and nearly all OVαβ TCR mice remained healthy throughout the course of the study, whereas 100% of the TCR transgenic RAG1 deficient became sick. Onset of disease was at an average of eleven weeks of age in all three susceptible mice.

RAG-sufficient mice carrying the transgenes for the OVαβ TCR or the MBPαβ TCR were monitored for a period up to twelve months. The incidence of spontaneous EAE was recorded from the onset of obvious hind leg paraparesis (gait disturbance). The animals were sacrificed when full hind leg paralysis was observed. Consistent with reported results, approximately 40% (4 of 10 mice, Fig. 5 A,B) of MBPαβ TCR mice developed EAE, with an average onset of approximately 11 weeks after birth [14], [31]. In contrast, only one OVαβ mouse was observed to develop EAE (Fig. 5 A,B). Onset of the disease in this mouse occurred 16 weeks after birth. A group of nine OVαβ TCR mice was monitored for a total of twelve months and more than 50 mice were monitored for a shorter period of time (∼6 months). Of this group, only one additional mouse (12 weeks old) showed any overt signs of the disease.

The greatly reduced frequency of EAE in the OVαβ TCR mice is most likely due to one, or a combination of, three factors. First, the lower frequency of OVαβ TCR T cells as compared to MBPαβ T cells may play a role; second, the lower affinity of the OVαβ TCR may make it inherently less encephalitogenic; or third, regulatory T cells (presumably CD4^+^CD25^+^ T cells) might be more effective at controlling the lower affinity OVαβ T cells. We sought to differentiate between these possibilities by studying the RAG1 deficient MBPαβ and OVαβ TCR transgenic mice.

It has been shown that if all endogenous TCR expression is removed from a mouse line carrying the transgenes for a different AC1-11/I-Au specific TCR, 100% of the mice develop EAE [15]. This result is due, at least in part, to the removal of residual CD4^+^CD25^+^ Treg cells that develop due to the expression of non-transgene encoded TCRs [16]. Therefore, we monitored RAG1 deficient mice carrying the transgenes for the high affinity MBPαβ TCR for signs of EAE. In contrast to RAG1 sufficient MBPαβ TCR mice, 100% of the MBPαβ TCR, RAG1 deficient mice (24 mice) developed EAE (Fig. 5 A, B). Interestingly, the RAG1 deficient mice developed EAE at the same age (11 weeks) as their RAG1 sufficient counterparts (Fig. 5A, Ref. [14], [31]. We also evaluated our RAG1 deficient OVαβ TCR transgenic mice. In sharp contrast to what we found in the OVαβ RAG1 sufficient mice, 100% (25/25 mice) of the OVαβ RAG1 deficient mice developed EAE (Fig. 5A, B). Remarkably, the age that the mice developed the disease was, on average, nearly identical to both the MBPαβ RAG1 sufficient and MBPαβ RAG1 deficient mice (Fig. 5A, B).

## Discussion

The underlying reasons for the onset of T cell mediated autoimmune diseases are complex and involve a variety of genetic and environmental factors. The presence of self-peptide:self-MHC specific T cells is, however, clearly essential. In general, a minimal affinity between self-MHC:self-peptide complexes and the TCR appears to be an important factor for the function T cells [32]. T cells that initiate autoimmune diseases, however, presumably have avidities for self-ligands that are closer to pathogen-derived antigen specific responses [33].

Autoimmunity is commonly thought to require high affinity “driver” T cell clones that initiate disease and cause inflammation [34]. This inflammation results in a DTH-like response that recruits macrophages and other non-specific T cells. The damage caused by the inflammation can also lead to epitope spreading, resulting in the activation of T cells specific for epitopes distinct from the disease-causing epitope. This secondary response, which appears to occur within the CNS, rather than peripheral sites [11], [35], may involve lower affinity T cell clones [36]. Self-antigen specific driver clones, therefore, must be present in all mouse strains that are susceptible to EAE. Nonetheless, EAE typically does not occur spontaneously; rather it must be stimulated by various immunization protocols. Even TCR transgenic mice that have nearly clonal populations of high affinity self-reactive T cells are completely [15] or at least partially [14] protected from the onset of spontaneous EAE. It has been previously shown for one of these models that when the TCR transgenes were introduced into mice deficient for RAG1 expression, EAE developed spontaneously [15]. Therefore, a minor population of lymphocytes that required RAG1 for development played a critical role in suppressing disease. Later, the suppressor cells were definitively determined to be CD4^+^ T cells expressing non-transgene encoded TCRs [16].

Our studies of a low-affinity MBP AC1-11 peptide/I-A^u^ specific TCR-transgenic model were designed to determine if the affinity of the TCR-MHC/peptide interaction played a role in the pathogenesis of EAE. We first characterized antigen specific T cells from mice carrying only the MBP TCRβ chain. Interestingly, our analysis revealed that the TCR repertoire of the AC1-11 MBP peptide reactive T cells in these mice was limited to only two different TCRs. These data imply that central tolerance is highly efficient at removing all other TCRs reactive for this ligand, but for unknown reasons, fails to remove thymocytes expressing these two TCRs. This TCRα chain was used to generate a new TCR transgenic mouse model, which was compared to an existing mouse model that expresses a related high affinity TCR.

It is not known how disease causing, self-reactive T cells escape intrathymic negative selection [37]. One possibility is that thymocytes expressing TCRs that have very high avidities for self-antigens actually are deleted due to the ectopic expression of peripheral “tissue-specific” proteins in the thymus [38], [39], [40], [41]. Indeed, it has previously been shown that mice lacking the last five exons of MBP (shiverer mice) mount T cell responses to additional MBP-derived epitopes [42], [43]. These data demonstrated that thymic expression of the MBP is directly responsible for tolerance of at least some MBP-reactive thymocytes. Tissue specific antigens, however, may be expressed at low levels and, therefore, may not be efficient at negative selection of lower affinity self-reactive clones [44]. A variant on this idea is that the form of the self-antigen expressed in the thymus is somehow distinct from the peripherally expressed antigen [11]. In the case of the shiverer mice, T cell responses against the AC1-11 MBP derived peptide in shiverer mice were similar to those in wild type mice suggesting that the relatively unstable I-A^u^:Ac1-11 complex is not efficient at driving clonal deletion [42], [43].

Whatever the exact mechanism of clonal escape might be, it was reasonable to hypothesize that thymocytes expressing the lower affinity OVαβ TCR would develop at least as efficiently those expressing the as the higher affinity MBPαβ TCR. Indeed, the lower affinity for self-antigen would arguably make the OVαβ TCR thymocytes less susceptible to clonal deletion. We found, however, that the low affinity OVαβ TCR was actually very poorly positively selected. There was no indication of negative selection (i.e. reduced cellularity, upregulation of CD69, etc.) in either mouse. Therefore, in contrast to finding that aberrant negative selection plays a significant role in allowing the escape of disease causing T cells, we find that positive selection is a major contributor. In other words, in our model system, high affinity, self-reactive T cells are efficiently positively selected, whereas, low affinity, less “dangerous” clones, fall by the wayside. This difference in positive selection may also account for the four-fold excess of high affinity T cell clones that we consistently find in the TCRβ only mice.

Consistent with previous studies [14], [31], in our analysis, we also found that ∼40% of mice carrying the transgenes for the high affinity MBPαβ TCR spontaneously developed EAE at approximately eleven weeks of age. This level of penentrance is consistent with what we have seen over several years of maintaining this mouse strain. Of the nine OVαβ TCR mice that we monitored for the complete twelve months, only one mouse developed EAE. Overall, approximately fifty OVαβ TCR mice were produced and were monitored for up to six months, with only one additional mouse showing clear signs of paralysis, which occurred at 12 weeks. The minimal number of sick OVαβ TCR mice is due either to the reduced affinity of the TCR and/or due to the reduced numbers/percentage of MBP specific T cells. Clearly the total number of potentially encephalitogenic T cells in the OVαβ TCR mice far exceeds what can be found in a wild type mouse, but, nonetheless, it is several fold fewer than the more disease prone MBPαβ TCR transgenic mice. Eliminating all endogenous TCRs by breeding to RAG1 deficient mice, however, demonstrated that both versions of the MBP specific T cells were likely kept in check by the presence of RAG-dependent regulatory cells. Based on previous work, these regulatory cells are, at least in part, CD25^+^CD4^+^ Tregs [16], [17]. These data, therefore, suggest one of two possibilities, which are not exclusive. First, the absolute ratio of Tregs to self-reactive T cells may be critical, with relatively subtle alterations resulting in disease. Alternatively, Tregs may be far more efficient at controlling self-reactive T cells with lower affinity TCRs. We anticipate that cell transfer studies will allow us to distinguish between these two mechanisms.

Rather surprisingly, the onset of EAE for the MBPαβ TCR mice on a wild type background and both the MBPαβ TCR and OVαβ TCR mice on a RAG1 deficient background was nearly identical, with a mean of eleven weeks. Even the two OVαβ TCR RAG1 sufficient mice in which disease was observed became sick around this timeframe (12 and 16 weeks). These data suggest that some initiating event, perhaps release of myelin basic protein due to apoptosis of nerve cells, reproducibly occurs soon after the mice enter adulthood. Perhaps this is related to the finding that self-reactive T cells contribute to neurogenesis [45]. Importantly, if RAG dependent cells, such as Tregs, are present during this “window of opportunity”, they are capable of preventing autoimmune disease.

Overall, our analysis of high and low affinity MBP Ac1-11 specific T cells reveals several important aspects concerning the onset of the autoimmune disease, EAE. First, the frequency of different clonotypes that are capable of recognizing the dominant Ac1-11 epitope appears to be very limited. Second, positive selection, rather than negative selection, appears to have a major impact on the frequency of different clonotypes. Third, regulatory cells appear to be very efficient at controlling self-reactive T cells expressing a low affinity TCR. And finally, potentially encephalitogenic T cells appear to have relatively short window of opportunity to cause disease. Intriguingly, if regulatory cells are present during this window of opportunity, which is around eleven weeks after birth, more often than not encephalitogenic T cell responses are suppressed and this suppression lasts a lifetime.
